# Residual gastric volume after 3 h of the ingestion of an oral supplement containing carbohydrates alone or associated with whey protein: a randomized crossover pilot study

**DOI:** 10.1186/s13741-022-00289-6

**Published:** 2022-12-27

**Authors:** Paulo Luiz Batista Nogueira, Mario Renato da Silva, Diana Borges Dock-Nascimento, José Eduardo de Aguilar-Nascimento

**Affiliations:** 1 UNIVAG Medical School, Varzea Grande and Health Sciences Postgraduate Department, Medical School, Federal University of Mato Grosso, Cuiabá, Brazil; 2 Department of Radiology, UNIVAG Medical School, Varzea Grande, Brazil; 3 Nutrition School and Health Sciences Postgraduate Department of Medical School, University of Mato Grosso, Cuiabá, Brazil

**Keywords:** Preoperative fast, Whey protein, Carbohydrate, Oral supplement, Gastric emptying

## Abstract

**Background:**

New formulas including a nitrogenous source to maltodextrin have been reported as preoperative beverages 2–3 h before anesthesia in the elective procedure. Whey protein is a potential candidate for the composition of this clear oral supplement. This study aimed to investigate the gastric residual volume (GRV) of healthy volunteers 3 h after the ingestion of an oral supplement containing carbohydrates (CHO) alone or combined with whey protein (WP).

**Methods:**

This crossover clinical trial design includes young, healthy male volunteers with normal body mass index. Magnetic resonance imaging (MRI) scan of the upper abdomen to measure the GRV was performed in the participants in three phases: (1) after a fasting period of 8 h; (2) immediately after the ingestion of 200 mL of a clear supplement containing: (2a) 10 g of WP and 54 g of CHO (74% glucose and 26% maltodextrin)–WP + CHO group or (2b) 12.5% maltodextrin (25 g)–CHO group; and (3) after 3 h of the ingestion of both types of supplements. A week interval was programmed between phases 2a and 2b.

**Results:**

There was no significant difference (*p* = 0.91; within-group comparison) of the mean ± SD of the GRV between phase 1 (WP + CHO: 23.45 ± 14.01; CHO: 25.03 ± 15.17 cm^3^; *p* = 0.78; between-groups comparison) and phase 3 (WP + CHO: 25.66 ± 9.31; CHO: 23.45 ± 13.58 cm^3^, *p* = 0.86; between-groups comparison). The GRV of phase 2 (WP + CHO: 206.43 ± 23; CHO: 203.99 ± 12.18 cm^3^; *p* = 0.82; between-groups comparison) was significantly greater (*p* < 0.01; within-group comparison) than both other two phases.

**Conclusion:**

The GRV after 3 h of the ingestion of either WP + CHO or CHO oral supplement returns to basal fast condition implying that gastric emptying after this interval of time is significantly completed.

**Trial registration:**

Registered and posted on the ClinicalTrials.gov public website with Identifier: NCT05573854.

## Background

One of the most important prescriptions of enhanced recovery after surgery protocols is the reduction of preoperative fasting time in opposition to the traditional recommendation of overnight fasting (Kuemmerli et al. 2022; De-Aguilar-Nascimento et al. 2017). This change from prolonged fast to only 2–3 h after the ingestion of 200–400 mL of carbohydrate (CHO)-enriched supplements is not only safe (Smith et al. 2014) but also recommended by various societies of anesthesiologists (Smith et al. 2011), surgeons, and nutrition (Weimann et al. 2021). A United States survey of the use of preoperative CHO-enriched supplements in colorectal enhanced recovery after surgery (ERAS) programs showed that a high adherence of this prescription (87.2%) in 78 hospitals (Singh et al. 2021). A long list of beneficial effects associated with the reduction of the preoperative fasting time with CHO beverages is reported ranging from the alleviation of thirst, hunger, and stress of the patient (Bilku et al. 2014) to metabolic benefits such as the improvement of insulin resistance (Faria et al. 2009; Ricci et al. 2022).

However, new formulas containing a nitrogenous source for preoperative beverages continue to evolve during the last years aiming to achieve better results. The addition of glutamine, antioxidants, and recently whey protein to CHO-enriched formula has been reported and has been associated with a greater reduction of insulin resistance (Ricci et al. 2022), a decrease of acute phase inflammatory response (Perrone et al. 2011) and increasing production of glutathione (Dock-Nascimento et al. 2012). The effect of whey protein-enriched preoperative drinks on the postoperative functional capacity of patients are awaited (Ho et al. 2020). Nevertheless, only a few studies have tested the gastric emptying of drinks containing whey protein in addition to CHO (De Aguilar-Nascimento et al. 2014). Therefore, a new study using appropriate tools to assess gastric residual volume (GRV) and designed to assess this purpose seems necessary. Thus, we aimed at investigating the GRV 3 h after the ingestion a formula containing maltodextrin alone or combined with whey protein in healthy volunteers.

## Methods

This is an experimental crossover clinical trial design. Inclusion criteria were being male; with normal nutritional status assessed by BMI (body mass index between 20 and 30 kg/m^2^); age ranging from 18 to 30 years old; healthy with no acute disease during the 3 months before the study, and without having gastroparesis or other gastric motility disorders. All these conditions were assessed by a questionnaire containing questions about health status. We planned to exclude participants who did not complete the study; did not comply with the experiment procedures; or who consumed alcoholic beverages on the day before the study. We also excluded those who have metallic prosthesis of any kind, including any tiny metal fragments in the body or complaining of anxiety disorders indoors or phobia to magnetic resonance imaging (MRI).

### Study design

The data were collected in three phases. Firstly, after a fasting period of 8 h, the participants attended the Radiology Service of the Hospital de Cancer, Cuiaba, Brazil, to perform MRI images of the upper abdomen with the objective of measuring the fasting status GRV (phase 1). In this phase, adequate stomach motility was observed to exclude cases of possible gastroparesis that could interfere with gastric emptying and expected results. GVR (cm^3^) was evaluated by tracking each slice’s region of interest (ROI), forming a volume by the sum of all ROIs. In phase 2, which was immediately after phase 1 each participant while still inside the MRI exam room ingested 200 mL of one of these two clear supplements formula depending of the phase as follows: phase 2.1) 12.5% maltodextrin (25 g)–CHO group or phase 2.2) the intervention drink containing whey protein and carbohydrates as described below. All participants participated of phase 2.1 and phase 2.2. For that, a week interval was programmed between phase 2.1 and phase 2.2. Immediately after drinking the supplement (in phase 2.1 and phase 2.2), the individual was immediately positioned in the supine position and another MRI scan for measurement of GRV was done. Finally, phase 3 was performed after a 3-h pause after the ingestion of the oral supplements. A third MRI scan of the upper abdomen was done to measure again the GVR as previously described.

### Intervention oral supplement

The oral supplement used as an intervention for the study group (WP + CHO group) was Nutren Fresh (Nestlé, São Paulo, Brazil). This oral non-residual supplement (clear fluid; 698 mOsm/L) has 100% whey protein isolated from total proteins, with various vitamins (B1, B6, C, D, niacin, and folic acid) with a “lemon tea” flavor. In addition, the formula contains no lactose or lipids; is formulated with a volume of 200 ml, with a caloric density of 1.28 kcal/ml; protein: 10 g in 200 ml of the product (100% whey protein isolate); and carbohydrate: 54 g in 200 ml of the product (74% glucose syrup and 26% maltodextrin). For control within a week interval, all participants went through the three phases of the experiment using a supplement containing 25 g of maltodextrin plus 200 mL of water (12.5%, 50 kcal per 100 ml and 285 mOsm/L; CHO group).

### MRI protocol

All exams were performed using the same MRI equipment which was a Magnetom Essenza 1.5 T device; Siemens Healthcare (Brazil). Each volunteer was placed in a supine position, with a body coil (Body Matrix) to capture the signal. Cross-sectional acquisitions with 40 slices (perpendicular to the longitudinal axis of the body) were performed in echo-planar fast spin echo (HASTE) sequences with a slice width of 4.0 mm at 3.0 mm intervals. This STEAM sequence (TR [repetition time] 774 ms, TE [echo time] 92 ms) visualizes fluids with hypersignal as opposed to the low signal from adjacent organs. Each acquisition of a set of images was performed during 15 s of apnea. The images were captured by an industry technician with MRI experience and reviewed by a radiologist to confirm that the images covered the entire stomach. The GRV was assessed by tracking each slice’s region of interest (ROI), forming a volume by the sum of all ROIs. GRV measurements in milliliter were performed manually by tracing regions of interest on each slice using OsiriX MD ANVISA v.12.0.3 software and summing the slices to determine the total gastric volume and the results were entered into an Excel data sheet for statistical analysis.

### Statistical analysis

Categorical variables were summarized by absolute (*n*) and relative (%) frequencies. Continuous were expressed as means and 95% confidence interval (95%CI) or standard deviation (SD). ANOVA for repeated measures was used to compare GRV in the three phases. Additionally, paired and unpaired *t* tests were done to assure within and between groups comparisons and adjust possible inflation of alpha error. The value of *p* for within-measures and between-measures analysis was calculated for analysis. For all analyses, *p* < 0.05 was considered statistically significant. All statistical analyzes were performed using SPSS for Windows 20.0 software (SPSS, Chicago, IL, USA).

## Results

**Fig. 1:**
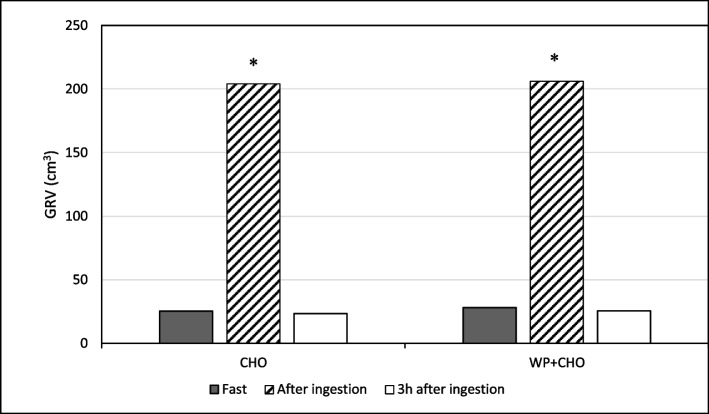
Mean gastric residual volume (GRV) during the three phases of the study in the two groups. Black bar: fasting (phase 1); diagonal stripes: after ingestion of 200 mL of the oral supplement (phase 2); and white bar: 3 h after ingestion of the oral supplement (phase 3). *p < 0.01 vs. Fast and 3 h after ingestion

The clinical and demographic characteristics of the participants showed they all were young and healthy (Table 1). The GRV observed in the three phases of the study can be seen in Table 2. Figure 1 shows the mean GVR in the two groups during the three phases of the study. There was no significant difference in the GRV between phase 1 (WP + CHO: 23.45 ± 14.01; CHO: 25.03 ± 15.17 cm^3^; *p* = 0.78; between-groups comparison) and phase 3 (WP + CHO: 25.66 ± 9.31; CHO: 23.45 ± 13.58 cm^3^, *p* = 0.86; between-groups comparison) in each of the two groups. The GRV of phase 2 (WP + CHO: 206.43 ± 23; CHO: 203.99 ± 12.18 cm^3^; *p* = 0.82; between-groups comparison) was significantly greater than both other two phases in each group (*p* < 0.01; within-group comparisons). Between-group comparisons showed no significant difference among groups in each phase of the experiment.

**Table 1:** Clinical and demographic characteristics of the participants

Variables	*n*
Age (years old)	20.50 (1.38)
Chronic health condition	
No	6 (100%)
Acute health condition	
No	6 (100%)
Use of medicines/drugs	
No	6 (100%)
Previous admission to the hospital	
No	6 (100%)
Tobacco	
No	6 (100%)
Body weight (kg)	68.50 (8.21)
BMI (kg/m^2^)*	21.62 (2.09)
Height (cm)	177 (3)

^*^BMI Body mass index. Data are mean (SD) or *N* (%)

**Table 2:** Distribution of the GVR during the three phases of the study in the two groups

Phase and group	Mean	SD	Median	Lower	Higher
**Fast (phase 1)**					
CHO	25.3	15.7	18.8	11.8	45.2
WP + CHO	28.2	24.0	19.2	8.4	70.7
**After ingestion (phase 2)**					
CHO	204.0*	12.2	204.7	190.7	220.3
WP + CHO	206.4*	23.5	205.6	168.3	239.5
**3 h after ingestion**					
CHO	23.5	13.6	19.6	11.0	42.2
WP + CHO	25.7	9.3	23.9	13.9	42.1

All between-group comparisons were NS (*p* > 0.05) in each phase. *p < 0.01 vs phase 1 and phase 2 in each group (within-group comparisons). Repeated-measures ANOVA

*CHO* Carbohydrate, *WP* Whey protein, *SD* Standard deviation

## Discussion

The overall findings of this randomized crossover study showed that the GRV returns to fast condition after 3 h of the ingestion of 200 mL of an oral supplement containing either CHO alone or combined with whey protein. The GRV as expected increased immediately after the ingestion but reverted to basal conditions 3 h after as can be seen in Fig. 1 and Table 2. This is relevant and suggested that oral supplements with whey protein can be safely emptied of the stomach after 3 h of ingestion which was similar to the findings with the supplement containing only CHO. Implications of these findings are that this formula is feasible to be used as a preoperative oral supplement preoperatively. The present data evoke the necessity of further investigation in selected patients who are candidates for elective procedures under anesthesia and who are allowed to receive preoperative oral supplement. Possible advantages of oral supplements containing whey protein in addition to CHO over drinks with CHO alone are not only in the positive nitrogen balance immediately after surgery but also in the positive effects on glucose metabolism and acute phase reaction to trauma (Ricci et al. 2022).

The emptying of food from the stomach into the small intestine is the result of a series of motor and secretory events through complex neuromuscular coordination with inhibitory and excitatory components, all of which are highly coordinated (Du et al. 2018). It is important to highlight that the continuous process of saliva swallowing and gastric fluid secretion always causes a basal GRV, even in a fasting state, with volumes ranging from 19 to 50 ml in physiological normal individual variations (Maltby 2006; Lobo et al. 2009). In agreement, our study showed that after an overnight fast the GRV was approximately 25 cm^3^ which is normal among healthy volunteers. The return of GRV to the basal condition in a crossover study like this is important and relevant because each individual is compared with himself. In addition, all volunteers drank during phase 2 with a week interval a control supplement (maltodextrin) and the intervention drink containing WP. Other small studies testing gastric emptying after oral supplementation with various amino acids and proteins have previously reported the safety of these supplements to be used 3 h before anesthesia (Dock-Nascimento et al. 2012; Perrone et al. 2011; Ho et al. 2020). In accordance, the guidelines of the Brazilian Society of Anesthesia recommend with a low grade the use of glutamine or whey protein 3 h before the induction of anesthesia in elective procedure in individuals without any contraindication for the abbreviation of preoperative fasting (Veloso et al. 2019).

However, these findings have limitations. It is a pilot study and we included only a few healthy young volunteers. Nevertheless, the overall findings allow us to conclude that the GRV, assessed by MRI, in healthy young volunteers after 3 h of the ingestion of 200 ml of an oral supplement containing carbohydrates associated with whey proteins and vitamins is similar to the GRV after overnight fast implying that it is safe for adequate gastric emptying. These data are in accordance with recent literature and urge further investigations with the use of this formula of oral supplements in patients who are candidates for elective surgical procedures.

## Conclusion

The GRV after 3 h of the ingestion of either WP + CHO or CHO oral supplement returns to basal fast condition implying that gastric emptying after this interval of time is significantly completed.

## Data Availability

All data generated or analyzed during this study are included in this published article.

## Abbreviations

GRV: Gastric residual volume
CHO: carbohydrate
ERAS: Enhanced recovery after surgery
BMI: Body mass index between 20 and 30 kg/m^2^
MRI: Magnetic resonance imaging
ROI: Region of interest magnetic resonance imaging
SD: Standard deviation

## References

[CR1] Bilku DK, Dennison AR, Hall TC, Metcalfe MS, Garcea G (2014). Role of preoperative carbohydrate loading: a systematic review. Ann R Coll Surg Engl.

[CR2] De-Aguilar-Nascimento JE, Caporossi C, Metelo JS, Tanajura GH, Canevari-de-Oliveira M, da Cunha CR (2014). Safe intake of an oral supplement containing carbohydrates and whey protein shortly before sedation to gastroscopy; a double-blind, randomized trial. Nutr Hosp.

[CR3] De-Aguilar-Nascimento JE, Salomão AB, Waitzberg DL, Dock-Nascimento DB, Correa MITD, Campos ACL (2017). ACERTO guidelines of perioperative nutritional interventions in elective general surgery. Rev Col Bras Cir.

[CR4] Dock-Nascimento DB, de Aguilar-Nascimento JE, MagalhaesFaria MS, Caporossi C, Slhessarenko N, Waitzberg DL (2012). Evaluation of the effects of a preoperative 2-hour fast with maltodextrine and glutamine on insulin resistance, acute-phase response, nitrogen balance, and serum glutathione after laparoscopic cholecystectomy: a controlled randomized trial. JPEN J Parenter Enteral Nutr.

[CR5] Du YT, Piscitelli D, Ahmad S, Trahair LG, Greenfield JR, Samocha-Bonet D (2018). Effects of glutamine on gastric emptying of low and high-nutrient drinks in healthy young subjects—impact on glycemia. Nutrients.

[CR6] Faria MS, de Aguilar-Nascimento JE, Pimenta OS, Alvarenga LC, Dock-Nascimento DB, Slhessarenko N (2009). Preoperative fasting of 2 hours minimizes insulin resistance and organic response to trauma after video-cholecystectomy: a randomized, controlled, clinical trial. World J Surg.

[CR7] Ho CY, Ibrahim Z, Abu Zaid Z, Mat Daud Z, MdYusop NB (2020). Fast-track- recovery surgery with a whey-protein-infused carbohydrate-loading drink pre-operatively and early oral feeding post-operatively among surgical gynaecological cancer patients: study protocol of an open-labelled, randomized controlled trial. Trials..

[CR8] Kuemmerli C, Tschuor C, Kasai M, Alseidi AA, Balzano G, Bouwense S (2022). Impact of enhanced recovery protocols after pancreatoduodenectomy: meta-analysis. Br J Surg..

[CR9] Lobo DN, Hendry PO, Rodrigues G, Marciani L, Totman JJ, Wright JW (2009). Gastric emptying of three liquid oral preoperative metabolic preconditioning regimens measured by magnetic resonance imaging in healthy adult volunteers: A randomized double-blind, crossover study. Clin Nutr.

[CR10] Maltby JR (2006). Fasting from midnight–the history behind the dogma. Best Pract Res Clin Anaesthesiol.

[CR11] Perrone F, da-Silva-Filho AC, Adôrno IF, Anabuki NT, Leal FS, Colombo T (2011). Effects of preoperative feeding with a whey protein plus carbohydrate drink on the acute phase response and insulin resistance. A randomized trial. Nutr J..

[CR12] Ricci C, Ingaldi C, Alberici L, Serbassi F, Pagano N, De Raffele E (2022). Preoperative carbohydrate loading before elective abdominal surgery: A systematic review and network meta-analysis of phase II/III randomized controlled trials. Clin Nutr.

[CR13] Singh SM, Liverpool A, Romeiser JL, Miller JD, Thacker J, Gan TJ (2021). A U.S. survey of pre-operative carbohydrate-containing beverage use in colorectal enhanced recovery after surgery (ERAS) programs. Perioper Med (Lond)..

[CR14] Smith I, Kranke P, Murat I, Smith A, O'Sullivan G, Søreide E (2011). Perioperative fasting in adults and children: guidelines from the European Society of Anaesthesiology. Eur J Anaesthesiol.

[CR15] Smith MD, McCall J, Plank L, Herbison GP, Soop M, Nygren J (2014). Preoperative carbohydrate treatment for enhancing recovery after elective surgery. Cochrane Database Syst Rev..

[CR16] Veloso KB, Pires OC, Oliveira SS, Nunes RR. Oral fast reduction and new oral supplements. In: Brandão JCM et al, editors. Medicina Perioperatória e Anestesia. Brazilian Society of Anesthesiologists. 2019. p. 59–68. https://saes.org.br/images/meta/0f132de0-3693-4884-87be-a2618a62c884/133/medicina-perioperato-ria-e-anestesia.pdf of subordinate document. Accessed 12 Oct 2022.

[CR17] Weimann A, Braga M, Carli F, Higashiguchi T, Hübner M, Klek S (2021). ESPEN practical guideline: clinical nutrition in surgery. Clin Nutr.

